# Molecular and Cellular Mechanisms of Plant Responses to Heavy Metal Stress in Mining-Impacted Environments

**DOI:** 10.3390/plants15071045

**Published:** 2026-03-28

**Authors:** Mădălina F. Ioniță, Emilia C. Dunca, Sorin M. Radu

**Affiliations:** 1 Department of Environmental Engineering and Geology, Faculty of Mining, University of Petrosani, 332006 Petrosani, Romania; madalina-flaviaionita@upet.ro; 2 Department of Mechanical, Industrial and Transport Engineering, University of Petrosani, 332006 Petrosani, Romania

**Keywords:** heavy metal stress, mining-impacted environments, plant molecular responses, oxidative stress, metal detoxification mechanisms, stress signalling, chronic metal exposure

## Abstract

Heavy metal contamination associated with mining activities is a major source of abiotic stress for plants, strongly affecting plant physiology, growth and survival in contaminated environments. Due to their non-biodegradable nature and long-term bioavailability, heavy metals persist in soils affected by mining activities, exposing plants to chronic stress conditions that require the activation of coordinated cellular and molecular response mechanisms to limit toxicity and maintain internal homeostasis. This review synthesises and critically analyses current knowledge on the molecular and cellular mechanisms governing plant responses to heavy metal stress in mining-affected environments. Key processes involved in metal uptake and transport, redox imbalance and oxidative stress generation, antioxidant defence systems, and molecular detoxification mechanisms, including metal chelation, subcellular compartmentalisation, and gene expression regulation, are discussed. Particular attention is paid to cellular signalling pathways that mediate plant adaptation to prolonged exposure to complex metal mixtures. Emphasis is placed on integrating molecular-level knowledge with the specific context of mining sites, highlighting the limitations of extrapolating results obtained under controlled experimental conditions to naturally contaminated environments. This perspective integrates molecular mechanisms with the geochemical realities of mining sites, providing a solid basis for the development of effective phytoremediation strategies and the optimisation of plant species selection.

## 1. Introduction

Recent advances in plant molecular sciences have demonstrated that responses to heavy metal stress are regulated by complex networks of cellular and molecular processes [1,2]. Metal absorption and transport are controlled by specific families of membrane transporters, and excessive accumulation induces redox imbalance and the generation of reactive oxygen species, with direct effects on cellular structures and metabolic functions. In parallel, plants activate detoxification mechanisms, including chelation by phytochelatins and metallothioneins, vacuolar compartmentalisation, and adaptive changes in the cell wall [3]. These processes are supported by the coordinated regulation of gene expression and stress-associated signalling pathways.

Most of the mechanisms described come from controlled experimental models, which do not fully reflect the complexity of chronic polymetallic exposure in mining sites. Systematic confirmation under real polymetallic conditions is still limited [4,5].

In this context, this paper synthesises and critically analyses the molecular and cellular mechanisms involved in plant responses to metal stress, with a focus on environments affected by mining [6]. Correlating molecular processes with specific geochemical conditions highlights how metal speciation, soil heterogeneity, and multimetallic interactions shape cellular responses under chronic exposure conditions [7].

The novelty of the approach lies in its explicit consideration of realistic environmental conditions by: (i) distinguishing between acute metal stress and chronic exposure specific to mining environments; (ii) assessing the influence of geochemical variability on molecular regulation; (iii) contextualising detoxification and redox regulation mechanisms in prolonged exposure; and (iv) identifying gaps that limit the extrapolation of laboratory results to actual contaminated sites. This perspective provides a coherent framework for interpreting plant responses to metal stress and for informing risk assessment and phytoremediation strategies.

The manuscript emphasises mechanistic interpretation in the context of chronic polymetallic exposure. Rather than reiterating established molecular pathways, it critically assesses the ecological relevance, stability, and adaptive significance of these mechanisms in contexts of long-term selective pressure.

**Figure 1:**
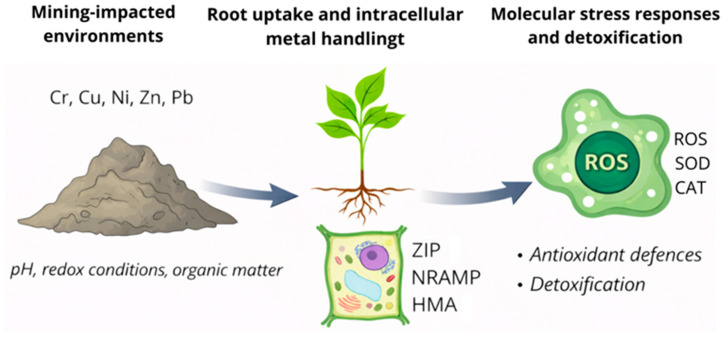
Conceptual framework summarising the relationships between heavy metal contamination in mining-impacted environments and plant cellular and molecular responses, encompassing metal uptake and transport pathways, redox imbalance and oxidative stress, molecular detoxification mechanisms, and stress-induced gene expression and signalling. The pathways described are mainly supported by mechanistic laboratory studies, with relatively limited validation under chronic polymetallic field conditions.

Figure 1 illustrates the conceptual framework of this analysis, highlighting the links between heavy metal contamination in mining-affected environments and the main cellular and molecular response pathways discussed in the following sections.

## 2. Literature Search and Evidence Classification Strategy

This review was conducted as a structured narrative synthesis designed to ensure transparency in the literature selection while allowing integrative analysis across heterogeneous experimental systems and mining-related environmental contexts.

### 2.1. Data Sources and Reference Management

Peer-reviewed publications were retrieved from Web of Science Core Collection, Scopus, and PubMed, complemented by additional searches in Google Scholar. The literature search focused mainly on studies published after 2000, reflecting advances in plant molecular biology, redox regulation, and environmental geochemistry. Earlier foundational studies were included where necessary to contextualise core mechanisms of metal homeostasis, detoxification, and stress signalling.

All references were imported and organised using Zotero, which enabled duplicate removal, thematic categorization of studies, and consistent citation management throughout the manuscript.

Search queries combined terms such as “heavy metal stress”, “mining soils”, “metal homeostasis”, “reactive oxygen species”, “phytochelatins”, “metallothioneins”, “multimetal exposure”, and “plant adaptation”, applied using Boolean operators.

### 2.2. Study Selection Criteria

Eligible studies were peer-reviewed research articles or high-quality reviews addressing molecular, cellular, physiological, or geochemical aspects of plant responses to heavy metal or metalloid exposure.

Particular attention was given to studies investigating redox regulation, reactive oxygen species (ROS) signalling, antioxidant systems, metal transport and homeostasis, thiol-mediated detoxification mechanisms, and adaptive responses under chronic polymetal exposure in mining environments.

Both controlled laboratory experiments and field investigations conducted in mining-affected environments were considered to ensure balanced representation of mechanistic insights and environmentally relevant responses.

Studies lacking clearly defined exposure conditions or not assessing plant responses were excluded.

### 2.3. Evidence Classification

To differentiate levels of environmental complexity, the selected literature was organised into three categories:

Class I—controlled single-metal laboratory studies;

Class II—semi-controlled soil-based experiments;

Class III—field investigations conducted under chronic polymetal exposure in mining environments.

This classification allows clear distinction between mechanistic responses observed under simplified experimental conditions and adaptive processes occurring in environmentally complex mining systems.

Given the heterogeneity of experimental designs, exposure scenarios, and environmental conditions, an integrative conceptual synthesis was adopted, as statistical aggregation would not adequately reflect the biological and geochemical variability of the included studies.

## 3. Stress Caused by Heavy Metals in Mining-Affected Environments: Environmental Context

Mining activities are one of the main anthropogenic sources of heavy metal contamination at both regional and global levels, through the generation and disposal of large volumes of solid and liquid waste [3,8]. Mining residues, tailings ponds and areas adjacent to mining operations are environments characterised by high concentrations of potentially toxic elements resulting from ore extraction, crushing and processing activities. Metals can occur in both primary and secondary forms, generated by alteration and oxidation processes, resulting in complex geochemical dynamics and variable bioavailability with a direct impact on cellular responses [9,10].

Unlike natural or agricultural soils, environments affected by mining are often characterised by extreme pedological and geochemical properties, which amplify the stress exerted on plants. Extremely low or high pH values, redox potential fluctuations, low organic matter content and unstable physical structures directly influence the mobility of metals and their interactions with the solid and liquid phases of soils [5,11]. Under oxidising conditions, certain metals may become more mobile and bioavailable, while under reducing conditions they may be temporarily immobilised but remain susceptible to remobilisation following environmental changes [12]. This variability creates a dynamic exposure context in which plants are subjected to fluctuating metal stress that cannot be reliably predicted based on total metal concentrations, highlighting the need to interpret metal stress in relation to mobilisation processes, speciation and cellular interactions.

The main heavy metals associated with mining activities and their predominant cellular and molecular effects on plants are summarised in Table 1, which illustrates the relationship between sources of contamination and affected biological targets.

A defining feature of metal stress in mining environments is simultaneous exposure to complex mixtures of heavy metals, rather than a single toxic element [13,14]. Interactions between metals can have synergistic or antagonistic effects, influencing their absorption, transport and toxicity in plants. For example, competition between metal ions for the same membrane transporters or intracellular binding sites can significantly alter biological responses compared to exposure to a single metal, simultaneously affecting metal transport, competition for binding sites and the regulation of metal stress-related gene expression [15,16]. In addition, the presence of essential metals in excessive concentrations can induce nutritional imbalances, further exacerbating the physiological stress of plants.

Plants growing in such environments are subject to chronic long-term exposure to metal stress conditions, which leads to the selection of adaptation strategies that are distinct from those observed in short-term laboratory experiments, reflecting long-term cellular and molecular adaptation processes [17,18]. These strategies may include restricting metal uptake at the root level, preferential redistribution of metals to specific cellular compartments, or activation of efficient detoxification mechanisms [19]. At the same time, metal stress is often accompanied by other abiotic stress factors, such as water deficit, salinity or nutrient deficiencies, leading to the overlap and interaction of multiple cellular and molecular response pathways [20].

From a molecular research perspective, mining environments provide an extremely relevant but still insufficiently explored framework for understanding plant responses to metal stress. Most current knowledge comes from studies conducted on artificial soils or controlled nutrient solutions that fail to reproduce the chemical and physical complexity of actual mining sites. This discrepancy limits the extrapolation of experimental results to field conditions and highlights the need to integrate the environmental context into the interpretation of molecular mechanisms [13]. Understanding how geochemical factors specific to mining environments modulate plant cellular and molecular responses is essential for realistic impact assessment and the development of effective management and intervention strategies based on molecular mechanisms.

Mechanistic links between geochemical variability and molecular regulation soil geochemical parameters, such as pH and redox potential (Eh), directly influence heavy metal speciation, mobility and membrane transport affinity, thus acting as upstream modulators of molecular responses to stress in plants [9,10]. The acidification frequently observed in mining residues increases the solubility and bioavailability of Cd^2+^ and Zn^2+^ [21], potentially increasing the induction of ZIP and HMA transporters and altering the kinetics of metal absorption. In contrast, fluctuations in redox potential can alter the valence states of redox-active metals such as Fe and Cu, thereby influencing intracellular ROS generation and downstream antioxidant signalling cascades [2,4].

These geochemically driven changes in metal speciation and bioavailability not only alter exposure intensity, but also actively shape transporter regulation, redox homeostasis, and gene expression patterns. Consequently, pH and redox variability in soils affected by mining should be considered an integral component of the regulatory network governing plant molecular responses to chronic polymetallic stress.

## 4. Absorption and Transport of Heavy Metals in Plants

The absorption and transport of heavy metals in plants are critical steps that determine both the extent of toxic element accumulation and the severity of physiological and molecular responses induced by metal stress [22]. In mining soils, metal absorption is determined not only by total concentrations, but especially by pH, redox potential, speciation, and ionic competition [23]. These factors control bioavailability and modulate transporter expression, directly influencing plant tolerance to metal stress.

Absorption and transport mechanisms have been well characterised in controlled experimental systems, but their dynamics in chronic polymetallic exposure remain insufficiently documented. Consequently, their interpretation in mining soils must be carried out with caution.

### 4.1. Heavy Metal Absorption at the Root–Soil Interface

The root system is the main point of entry for heavy metals into plants, with absorption occurring mainly through membrane transport systems physiologically dedicated to the acquisition of essential nutrients. Essential metals such as Cu, Zn and Ni can become toxic when present in excessive concentrations, while non-essential metals can exploit the same transport pathways due to their chemical similarity to nutrient ions [24]. In mining environments, where the soil solution often contains complex mixtures of metals, competition for transporters is a key determinant of selective or cumulative uptake.

Absorption processes are modulated by factors such as rhizosphere pH, redox potential and microbiological activity, which influence the chemical forms and mobility of metals. Under acidic or oxidising conditions, certain metals may become more bioavailable, increasing their flux to root cells [10,25]. At the same time, root exudates and local changes in rhizosphere chemistry can contribute either to the mobilisation of metals or, conversely, to their immobilisation, directly affecting the amount of metal absorbed by the plant and the activation of cellular pathways involved in the perception and response to metal stress. The selectivity and efficiency of these absorption processes depend largely on the expression levels and specificity of the membrane transporters involved in metal acquisition at the root cell level.

In soils affected by mining, metal uptake at the root–soil interface is strongly influenced by geochemical parameters such as pH, redox potential (Eh) and ionic competition within complex metal mixtures [26]. For example, the acidic conditions frequently observed in mining residues increase the mobility of Cd^2+^ and Zn^2+^, potentially increasing transporter-mediated influx compared to neutral laboratory media [27]. In contrast, fluctuating redox conditions can alter Fe and Cu speciation, indirectly modifying uptake kinetics and subsequent intracellular signalling. Such dynamic soil conditions are rarely replicated in hydroponic experiments, highlighting a limitation in directly extrapolating laboratory-derived uptake mechanisms to field environments.

### 4.2. Metal Transporters Involved in Cellular Uptake and Translocation

At the molecular level, metal absorption and transport are mediated by specific families of membrane transporters whose expression and activity are strictly regulated according to metal availability, cellular redox status, and the physiological state of the plant. Members of the ZIP family (ZRT/IRT-type proteins) are mainly involved in the transport of Zn^2+^, Fe^2+^ and Mn^2+^, but can also facilitate the absorption of other metals in conditions of excess [28]. Transporters belonging to the NRAMP (Natural Resistance-Associated Macrophage Proteins) family play an important role in the cellular and intracellular transport of bivalent metals, contributing to their redistribution between cellular compartments [8,29].

Heavy metal Aâdenosine triphosphate (ATPases) (HMAs) participate in the active translocation of metals across membranes, using ATP as an energy source. These transporters contribute both to the long-distance transport of metals to the aerial parts of plants and to detoxification processes through vacuolar sequestration. Under conditions of chronic metal stress, the expression of HMA transporters is frequently altered, reflecting adaptive modulation in response to prolonged exposure to heavy metals [18,30].

Oxidative stress models require contextualisation in relation to the geochemical variability of mining sites. Several original field investigations, conducted directly on metallophyte populations inhabiting contaminated mining substrates, provide empirical support for the stable reconfiguration of transporters under conditions of chronic exposure. In naturally established populations of *Arabidopsis halleri* on Zn/Cd-rich mining soils, genomic amplification and constitutive overexpression of HMA4 have been repeatedly documented, correlating with an increased capacity for metal translocation from roots to shoots and sustained tolerance under field conditions [31]. Similarly, comparative transcriptomic analyses of field-collected *Noccaea caerulescens* ecotypes from polymetallic mining areas reveal constitutive activation of specific metal transporter networks compared to uncontaminated populations [17]. These findings suggest that chronic environmental selection pressure may stabilise certain transporter hierarchies at the population level, supporting adaptive metal homeostasis beyond transient responses induced in the laboratory. However, systematic comparisons between species under heterogeneous mining conditions remain relatively limited.

To facilitate a structured comparison between major transporter families and to distinguish mechanistic evidence derived from acute experimental systems from observations reported under chronic mining exposure conditions, the main characteristics and validation status of major metal transporter families are summarised in Table 2.

The comparative presentation in Table 2 highlights the discrepancy between detailed characterisation in the laboratory and relatively limited field validation of transporter regulation under conditions of chronic exposure to polymetals.

### 4.3. Long-Distance Transport and Distribution of Metals in Plants

After absorption at the root, heavy metals can be transported to the aerial organs through the xylem, either in free ionic form or complexed with organic ligands. The distribution of metals in plants is a finely tuned process that influences both accumulation in leaves and the protection of sensitive tissues [32]. In mining environments, where exposure is usually chronic, plants can develop strategies to restrict the translocation of metals to the aerial parts, thereby reducing the toxic effects on photosynthetic processes and preserving the cellular integrity of aerial tissues.

Most current knowledge on xylem loading, phloem redistribution, and long-distance metal transport comes from controlled experimental systems. Field validation under mining conditions remains relatively rare [1,29]. Under conditions of chronic exposure to polymetals, plants may exhibit an adaptive restriction of translocation from root to shoot, a trait frequently associated with phytostabilisation strategies observed in metallophytic species. However, the molecular regulation of such restriction under heterogeneous field conditions is not yet sufficiently elucidated.

In some cases, metals can also be redistributed through the phloem, particularly in plants exhibiting tolerance or hyperaccumulation traits. These processes depend on interactions between transporters, organic ligands and hormonal regulatory mechanisms, highlighting the complexity of plant responses to metal stress.

### 4.4. Membrane Transporters in Heavy Metal Uptake and Redistribution

At the molecular level, metal uptake and transport are mediated by specific families of membrane transporters whose expression and activity are tightly regulated according to metal availability, cellular redox status, and the physiological condition of the plant [8,19,33]. Members of the ZIP (ZRT/IRT-like Proteins) family are predominantly involved in the transport of Zn^2+^, Fe^2+^, and Mn^2+^ but may also facilitate the uptake of other metals under conditions of excess. Transporters belonging to the NRAMP (Natural Resistance-Associated Macrophage Proteins) family play an important role in the cellular and intracellular transport of divalent metals, contributing to their redistribution among cellular compartments [34].

Heavy metal ATPases (HMAs) are involved in the active translocation of metals across membranes, using ATP as an energy source. These transporters are essential both for the long-distance transport of metals to aerial plant parts and for metal detoxification through vacuolar sequestration [35,36]. Under conditions of chronic metal stress, the expression of HMA transporters is frequently altered, reflecting plant adaptation to prolonged exposure to heavy metals.

However, most functional characterisations of these transporters are derived from plants exposed to single metals under controlled nutrient conditions, and their regulation and specificity under chronic, multimetal exposure typical of mining environments remain insufficiently validated.

### 4.5. Subcellular Compartmentalisation as a Key Detoxification Strategy

Subcellular compartmentalisation is a fundamental strategy by which plants limit heavy metal toxicity. Once inside the cell, metals can be sequestered in vacuoles, bound to phytochelins or metallothioneins, or associated with the cell wall, thus reducing their interaction with sensitive structures such as mitochondria and chloroplasts [25,34]. These mechanisms allow essential metabolic functions to be maintained by lowering the concentrations of free metals in the cytosol and limiting toxic interactions with vulnerable cellular components.

The importance of subcellular compartmentalisation is particularly evident in mining environments, where plants are exposed to continuous and multimetallic stress. The ability to efficiently direct metals to compartments with lower metabolic activity is a determining factor for plant survival and long-term adaptation [37]. In this context, metal transport and compartmentalisation cannot be analysed independently, but must be understood as integrated components of a complex cellular response to heavy metal stress.

Under conditions of chronic exposure typical of mining environments, efficient subcellular compartmentalisation is not only an acute detoxification response, but also a long-term adaptation strategy that contributes to cellular homeostasis under conditions of persistent metal pressure. Vacuolar sequestration is well characterised experimentally, but field data on its stability under chronic exposure are still fragmentary.

**Figure 2:**
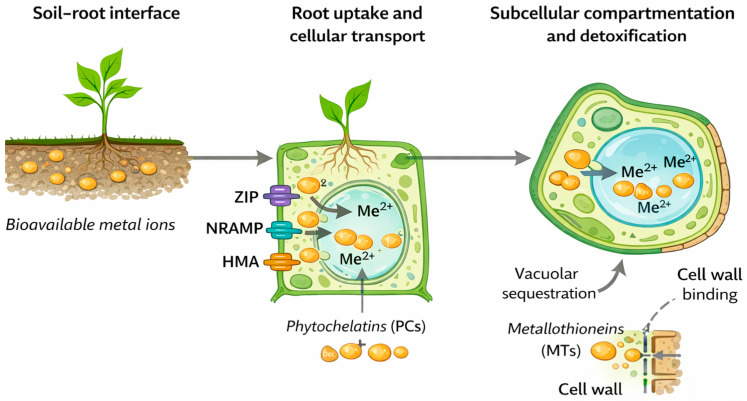
Conceptual illustration of heavy metal uptake, transport, and subcellular compartmentation in plants exposed to chronic metal stress in mining-impacted environments, emphasising the integration of uptake processes, long-distance transport, and intracellular detoxification strategies. Most of the mechanistic components represented are derived from controlled experimental systems, while long-term validation in the field under heterogeneous mining conditions remains relatively rare.

The main pathways involved in heavy metal uptake at the soil–root interface, their transport within the plant, and subcellular compartmentalisation are summarised schematically in Figure 2.

### 4.6. Environmental Context and Mechanistic Limitations

Environments affected by mining introduce dynamic and heterogeneous exposure conditions that can substantially modulate sorption and transport processes. Therefore, mechanistic knowledge derived from simplified laboratory systems requires cautious interpretation when applied to chronically contaminated field soils [38].

To provide a structured overview of the current level of mechanistic support and environmental validation for the sorption, transport, and compartmentalization processes discussed in this section, Table 3 summarises the predominant type of evidence and degree of field confirmation.

As Table 3 shows, validation under field conditions is mainly concentrated on natural metallophytes, while for most transporters characterised in the laboratory there is a lack of systematic evidence in real polymetallic contexts. This gap limits the ability to extrapolate and justifies the need for in situ molecular studies [39].

## 5. Cellular Toxicity and Redox Imbalance Induced by Heavy Metals

Heavy metal-induced cellular stress is one of the most significant consequences of plant exposure to contaminated environments, particularly in areas affected by mining. Once metals enter plant cells, they can directly or indirectly interfere with essential metabolic processes, leading to disturbances in redox homeostasis and the initiation of oxidative stress [40]. The imbalance between the production of reactive oxygen species (ROS) and the ability of antioxidant systems to neutralise them is a central mechanism through which metal stress manifests itself at both the cellular and molecular levels, affecting not only cellular structural integrity but also redox-dependent signalling networks [41].

Most mechanistic knowledge of heavy metal-induced oxidative stress comes from short-term studies of exposure to a single metal conducted under controlled laboratory conditions. Although these investigations have been fundamental to elucidating redox imbalance and antioxidant activation pathways, relatively few studies have examined redox dynamics under conditions of chronic exposure to multiple metals, typical of environments affected by mining. Consequently, extrapolating laboratory-derived oxidative stress models to field conditions requires careful contextualisation.

### 5.1. Generation of Reactive Oxygen Species Under Heavy Metal Stress

Heavy metals can stimulate ROS generation through multiple mechanisms, including interference with electron transport chains in mitochondria and chloroplasts, activation of cyclic redox reactions, and disruption of electron transfer in respiratory and photosynthetic complexes. Under conditions of metal stress, increased production of superoxide radicals, hydrogen peroxide, and hydroxyl radicals leads to oxidative stress, which compromises cellular structural integrity [42]. The intensity of ROS generation is influenced by the type of metal, its chemical form, and the duration of exposure, as well as environmental conditions that modulate the bioavailability of the metal.

In mining environments, where plants are chronically exposed to mixtures of heavy metals, ROS generation can become a persistent process, amplifying toxic effects on cellular metabolism [22,41]. This sustained production of ROS is not only a secondary consequence of metal toxicity, but also acts as a signal that triggers cascades of molecular responses. Excessive ROS accumulation thus reflects both the direct toxic effects of metals and the disruption of cellular redox control mechanisms.

In soils affected by mining, ROS generation is not only a function of intracellular metal accumulation, but is modulated by environmental parameters that influence metal speciation. The acidic pH conditions characteristic of many mining residues increase the mobility of Cd^2+^ and Zn^2+^, raising the potent nd cellular oxidative pressure [27]. In contrast, fluctuating redox potential can temporarily immobilise certain metals, followed by episodic remobilisation events that generate oxidative bursts. Such dynamic exposure patterns are rarely replicated in hydroponic systems, where metal concentrations and speciation remain stable.

### 5.2. Redox Imbalance and Oxidative Stress at the Cellular Level

Redox homeostasis represents a delicate balance between oxidative and antioxidant processes, which is essential for the normal functioning of plant cells [14]. Exposure to heavy metals disrupts this balance by increasing the flow of free electrons and inhibiting enzymes involved in redox processes. Redox imbalance affects both cell integrity and redox-dependent signalling networks, directly influencing gene expression regulation and adaptation to metal stress.

Acute exposure induces rapid ROS accumulation and transient antioxidant activation. In contrast, chronic polymetallic exposure may cause stable adjustments of redox thresholds and ROS-dependent gene regulation [43]. Although such adaptations have been suggested in metallophytes, long-term redox dynamics under mining conditions remain insufficiently characterised.

Under conditions of metabolic stress, oxidative stress manifests itself through the peroxidation of cell membrane lipids, protein oxidation, and nucleic acid damage [44]. These changes compromise membrane integrity, enzymatic activity, and genomic stability, ultimately leading to growth inhibition and, in severe cases, programmed cell death. In mining environments, where fluctuations in pH and redox potential are common, the intensity of oxidative stress can vary significantly over time and space, amplifying the dynamic nature of cellular responses.

### 5.3. Heavy Metal-Activated Antioxidant Defence Systems

To counteract the harmful effects of oxidative stress, plants possess complex antioxidant defence systems that comprise both enzymatic and non-enzymatic components. Enzymes such as superoxide dismutase (SOD), catalase (CAT) and various peroxidases (POD) play an essential role in detoxifying ROS and maintaining redox homeostasis [45]. The activity of these enzymes is often intensified under metal stress conditions as part of a coordinated cellular antioxidant response. In addition to enzymatic antioxidants, low molecular weight antioxidant molecules, including glutathione, ascorbate and various phenolic compounds, contribute to buffering oxidative stress [46]. The role of glutathione is particularly important, as it participates in both ROS detoxification and the synthesis of phytoquelatins involved in metal chelation. In mining environments, the efficiency of antioxidant systems can determine differences in tolerance or sensitivity to metal stress between plant species or populations [4].

Although improved SOD, CAT, and peroxidase activities are consistently reported under laboratory conditions of metal exposure, field evidence from mining sites suggests greater variability in antioxidant responses, likely reflecting species-specific adaptation and interaction with additional abiotic stressors such as drought or nutrient limitation.

For example, *Brassica juncea* exposed to cadmium has been reported to exhibit increased SOD and CAT activities as part of an inducible antioxidant response under controlled conditions [47]. In contrast, populations of *Arabidopsis halleri* growing naturally on Zn-rich mining substrates exhibit enhanced glutathione-dependent antioxidant capacity associated with chronic exposure and long-term adaptation. Similarly, *Silene vulgaris* populations colonising polymetallic residues exhibit altered ROS scavenging enzyme profiles compared to uncontaminated populations, suggesting population-specific modulation of antioxidant networks under field conditions [17].

Cadmium is a representative example in this context, as Cd-induced antioxidant defence has been extensively documented and illustrates how ROS detoxification capacity contributes directly to tolerance under both experimental and environmentally relevant exposure scenarios [48].

### 5.4. Cellular Damage and Redox-Dependent Signalling

Although oxidative stress is commonly associated with cellular damage, reactive oxygen species also function as central signalling molecules involved in regulating plant responses to stress [32]. The biological outcome of ROS accumulation depends on their concentration, subcellular localization, and temporal dynamics. In the case of moderate and transient increases, ROS act as redox signals that activate adaptive signalling pathways and the expression of stress-responsive genes. In contrast, sustained ROS accumulation during prolonged exposure to heavy metals can overwhelm cellular buffering capacity, leading to oxidative damage and disruption of redox-regulated signalling networks [14].

The dual role of ROS as harmful agents and signalling molecules has been extensively documented in model systems subjected to short-term exposure to metals. However, under chronic exposure conditions typical of mining-affected environments, sustained oxidative stress can progressively remodel redox-sensitive signalling networks, potentially altering the amplitude, duration, and integration of ROS-dependent transcriptional responses. Such long-term modulation of signalling architecture remains insufficiently characterised under field conditions [10].

Under chronic exposure conditions, persistent oxidative stress can recalibrate redox signalling thresholds, altering the sensitivity and amplitude of ROS-dependent transcriptional responses.

**Figure 3:**
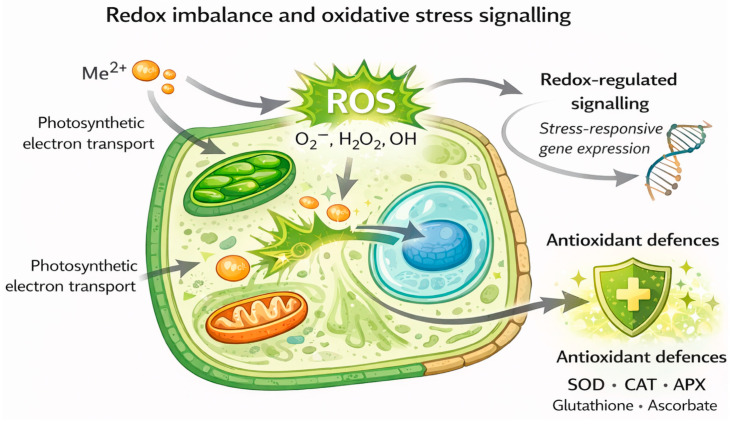
Conceptual illustration of redox imbalance and oxidative stress signalling in plant cells under chronic heavy metal exposure in mining-impacted environments, emphasising the interplay between ROS production, antioxidant defences, and redox-regulated signalling networks. The signalling pathways illustrated are mainly characterised in short-term laboratory exposure models, with limited direct in situ confirmation in mining-affected ecosystems.

The main sources of ROS generation, antioxidant mechanisms, and redox-dependent signalling pathways activated under metal stress conditions are schematically illustrated in Figure 3.

In soils affected by mining, fluctuations in pH and redox potential can alter metal speciation and, consequently, the dynamics of ROS generation compared to simplified laboratory systems [49]. For example, increased metal mobility under acidic conditions may intensify cellular redox imbalance, while reducing conditions may temporarily reduce bioavailability but create episodic oxidative bursts following reoxidation events. Such dynamic exposure patterns are rarely replicated under controlled experimental conditions.

### 5.5. Environmental Context and Evidence Limitations

Despite detailed mechanistic knowledge gained in controlled exposure systems, the applicability of oxidative stress models to mining-affected environments remains limited by environmental heterogeneity [50]. Chronic exposure to multiple metals, soil pH fluctuations, redox variability, and concomitant abiotic stress can substantially modulate redox dynamics and antioxidant regulation.

To facilitate systematic comparison of the strength of evidence in different exposure contexts, the current level of empirical support for the main redox and antioxidant mechanisms is summarised in Table 4.

## 6. Molecular Mechanisms of Detoxification and Tolerance in Plants Exposed to Heavy Metals

The survival of plants in environments contaminated with heavy metals depends on the activation of effective molecular detoxification mechanisms. These mechanisms limit the interaction of metals with sensitive cellular structures and allow the maintenance of essential metabolic functions [51]. In mining environments, characterised by chronic and multifactorial exposure, detoxification is not just a transient response, but defines the long-term tolerance threshold to metal stress.

Under chronic conditions, characteristic of mining residues, ATP-dependent detoxification mechanisms, such as sustained phytochelate synthesis and active vacuolar transport, involve high energy costs [36]. In nutrient-poor soils, plants may favour more energy-conservative strategies, such as apoplastic immobilisation or restriction of translocation to aerial organs. However, comparative assessments of these energy trade-offs under real field conditions remain limited.

### 6.1. Heavy Metal Chelation by Phytochelatins and Metallothioneins

Metal chelation is one of the most effective molecular detoxification mechanisms, whereby metal ions are bound to specific ligands and transformed into less toxic complexes. Phytochelatins are cysteine-rich peptides enzymatically synthesised from glutathione in response to heavy metal exposure [48,52]. Their ability to chelate metals such as Cd, Pb, Cu and Zn reduces the concentration of free metal ions in the cytosol and limits their interaction with sensitive molecular targets [53].

Cadmium represents one of the best-characterised models for understanding the integration of plant defence mechanisms involved in metal accumulation and tolerance. Recent studies have shown that Cd tolerance is not determined by a single detoxification pathway, but by the coordinated action of glutathione-dependent phytochelatin synthesis, metallothionein induction, antioxidant defence activation, and transporter-mediated sequestration or redistribution [34,43,48]. In Cd-exposed plants, the efficiency of tolerance depends on the balance between cytosolic chelation, vacuolar compartmentalisation, restriction of root-to-shoot translocation, and the maintenance of redox homeostasis [43,48]. In addition, progress in transcriptomic and multi-omic analyses has highlighted that Cd accumulation and tolerance are regulated through integrated defence networks involving stress-responsive genes, thiol metabolism, ROS signalling, and metal transporter expression [54]. These advances are particularly relevant for mining-affected environments, where chronic Cd exposure is often combined with other metals and with nutrient or water limitations, thereby requiring defence responses that remain stable under long-term environmental pressure [34,48].

Metallothioneins are another important class of metal-binding proteins, playing a dual role in the homeostasis of essential metals and the detoxification of toxic metals. Metallothionein expression is frequently induced under conditions of metal stress, and their tissue-specific distribution suggests distinct functions in cellular protection [55]. In mining environments, where plants are simultaneously exposed to multiple metals, the coordinated action of phytoquelatins and metallothioneins enhances the flexibility and efficiency of molecular detoxification responses [55,56].

Although phytoquelatin synthesis and metallothionein induction are consistently reported in acute laboratory experiments, their quantitative contribution to long-term metal tolerance in field-grown plants may vary substantially. In mining environments characterised by persistent exposure to multiple metals and nutritional imbalance, chelation capacity may be insufficient without coordinated sequestration and metabolic adjustment [57]. Field evidence from metallophyte populations suggests that chelation efficiency interacts with transporter regulation and compartmentalisation rather than functioning as an isolated detoxification mechanism.

### 6.2. Vacuolar Sequestration and Intracellular Compartmentalisation

Vacuolar sequestration of metals is a major detoxification strategy, allowing the isolation of metal ions from metabolically active cellular processes. The transport of metals or metal-ligand complexes into vacuoles is mediated by specific transporters located in the tonoplast, whose activity is strictly regulated at the molecular and transcriptional levels. Through this compartmentalisation, plants can tolerate high metal concentrations without compromising cytosolic or organelle functions.

The importance of vacuolar sequestration is amplified in mining environments, where prolonged exposure to metals requires long-term storage solutions. The ability to efficiently direct metals to vacuoles often distinguishes tolerant or hyperaccumulator species from sensitive ones, highlighting the central role of intracellular compartmentalisation in cellular and molecular adaptation to metal stress.

However, the efficiency of this process involves a significant energy cost, especially under conditions of chronic exposure. In mining-affected soils, where metal stress coexists with nutritional deficiencies, low organic matter content, and water stress, sustaining ATP-dependent transport to vacuoles can become a limiting factor [58]. In these contexts, the differences between tolerant metallophytes and unadapted species reflect not only their accumulation capacity but also the energy sustainability of compartmentalization mechanisms. Although the function of tonoplast transporters has been extensively characterised in controlled systems, long-term validation of sequestration dynamics under heterogeneous mining conditions remains insufficiently explored.

### 6.3. The Role of the Cell Wall in Metal Immobilisation

The cell wall of plants plays an important but often underestimated role in heavy metal detoxification. Due to its rich composition of polysaccharides, pectins, and functional groups capable of binding cations, the cell wall can act as a passive barrier with direct implications for ion fluxes and cellular metal distribution [59]. Metal fixation in the cell wall reduces the influx of metals into internal compartments and contributes to the attenuation of toxic effects.

Under conditions of chronic metal stress, the structure and composition of the cell wall may undergo adaptive changes that enhance its metal-binding capacity. These structural adjustments represent an important mechanism of cellular tolerance, particularly in mining environments, where metal concentrations can vary significantly in time and space [60,61].

In mining soils characterised by pH fluctuations and high metal concentrations, apoplastic immobilisation at the cell wall may represent an energy-conserving tolerance strategy compared to ATP-dependent intracellular sequestration. By limiting the influx of cytosolic metals at an early stage, cell wall binding can reduce the metabolic burden associated with prolonged detoxification [62]. Such immobilisation may be particularly advantageous for phytostabilising species adapted to nutrient-poor substrates. However, detailed molecular characterisation of adaptive changes in the cell wall under conditions of chronic field exposure remains relatively limited compared to laboratory investigations.

### 6.4. Integration of Detoxification Mechanisms and Metal Tolerance

Molecular detoxification mechanisms do not function in isolation but are integrated into a coordinated network of processes that include chelation, transport, compartmentalisation and gene expression regulation. Tolerance to heavy metals results from the coordination of these mechanisms, allowing plants to efficiently manage metal stress without compromising growth and development. In mining-affected environments, such integration is essential for long-term adaptation to persistent contamination [63].

Differences between species and populations reflect variations in the efficiency and regulation of detoxification mechanisms, and understanding these is essential for identifying tolerance-associated traits and optimising phytoremediation strategies [12].

The main molecular detoxification mechanisms contributing to plant tolerance to heavy metal stress and their integration at the cellular and physiological levels are summarised in Table 5.

From an applied and ecological perspective, differentiating between detoxification strategies is essential when designing phytoremediation strategies for sites affected by mining. Mechanisms that restrict translocation from roots to shoots and enhance apoplastic immobilisation are particularly relevant for phytostabilisation approaches aimed at limiting metal mobility and trophic transfer. In contrast, enhanced translocation capacity and chelation efficiency may favour phytoextraction strategies, although such approaches require careful assessment of ecological risks. It is important to note that the effectiveness of any of these strategies cannot be reliably inferred from mechanistic laboratory data alone and must be interpreted in conjunction with site-specific geochemical and environmental parameters.

## 7. Gene Expression and Stress Signalling Pathways in Response to Heavy Metal Exposure

Exposure of plants to heavy metals triggers profound changes in gene expression and activates complex cellular signalling networks that coordinate adaptive responses, tolerance mechanisms or, in severe cases, programmed cell death. Unlike the immediate responses associated with oxidative stress, gene expression regulation reflects medium- and long-term adaptation to metal stress, allowing plants to adjust their metabolism and cellular functioning under conditions of persistent exposure [63]. In mining environments, characterised by chronic and multifactorial stress, these regulatory mechanisms play an essential role in plant survival by reprogramming gene expression and fine-tuning cellular signalling networks.

The current framework for transcriptional regulation is derived mainly from acute experimental systems; stable reprogramming under chronic mining conditions is still poorly documented [64].

### 7.1. Transcriptional Regulation Under Heavy Metal Stress

At the molecular level, metal stress significantly alters gene expression profiles, affecting genes involved in metal transport, detoxification, antioxidant metabolism, and hormonal regulation [65]. Their activation or repression is controlled by transcription factors sensitive to redox status and signals generated by metal accumulation.

With prolonged exposure, plants can develop stabilised expression patterns associated with heavy metal stress tolerance. Genes encoding transporters, antioxidant enzymes, and proteins involved in chelation and compartmentalisation are frequently upregulated. This coordination reflects the need to maintain a balance between the absorption of essential metals and the limitation of excessive accumulation of toxic elements [27]. In heterogeneous mining environments, gene expression plasticity is a major adaptive advantage.

This is particularly evident under cadmium stress, where transcriptional regulation coordinates metal transport, thiol-mediated detoxification, antioxidant responses, and long-term adaptive adjustment of cellular defence networks [54].

Early transcriptional responses are characterised by rapid induction of defence genes under acute conditions. In contrast, chronic exposure may favour the establishment of semi-constitutive regulatory configurations associated with metabolic adjustment and long-term adaptation [66]. This transition from inducible responses to stabilised configurations may reflect the evolutionary adaptation of metallophyte populations subjected to persistent metal pressure. Evidence for the long-term stability of these transcriptional signatures under real mining conditions is still insufficiently supported by longitudinal studies.

### 7.2. The Role of Transcription Factors in Responses to Metal Stress

Transcription factors represent central nodes in the regulatory networks governing plant responses to metal stress. Various families of transcription factors are involved in the perception of stress signals and the activation of target genes associated with detoxification and tolerance [67]. These factors integrate information derived from redox state changes, hormonal signalling, and metal-induced metabolic disturbances, acting as key regulators of transcriptional responses.

Several families of transcription factors have been specifically associated with the regulation of heavy metal stress. Members of the bZIP family, such as bZIP19 and bZIP23, are key regulators of responses to zinc deficiency and excess, modulating transporter expression and metal homeostasis. WRKY and MYB transcription factors have been implicated in cadmium-induced stress signalling and antioxidant regulation, while members of the NAC family are frequently associated with the modulation of oxidative stress and programmed cell death in cases of prolonged metal exposure [54]. These families illustrate the functional diversity of transcriptional regulators that integrate metal perception with detoxification and redox signalling pathways.

Although numerous families of transcription factors have been implicated in responses to heavy metal stress, their functional characterisation comes mainly from overexpression studies in the laboratory or loss-of-function (gene knockout) studies conducted under simplified exposure conditions [68]. Direct evidence demonstrating the differential regulation of specific transcription factor networks in plants growing naturally on mining sites remains relatively scarce. Consequently, caution is warranted when extrapolating transcriptional regulatory hierarchies identified in model systems to complex, polymetallic field environments.

Under conditions of chronic metal stress, the expression and activity of transcription factors may be adjusted to favour cellular protection mechanisms at the expense of optimal physiological performance, reflecting an adaptive molecular trade-off. This transcriptional reprogramming constitutes a survival strategy whereby plants prioritise persistence over maximum growth [69]. The role of transcription factors in mediating these compromises is particularly relevant in mining environments, where metal stress is persistent and frequently combined with other abiotic stress factors.

### 7.3. Stress Signalling Pathways Activated by Heavy Metals

Cellular signalling plays a crucial role in coordinating molecular responses to metal stress. Metal-activated signalling pathways include calcium-dependent networks, protein kinase cascades, and redox-sensitive mechanisms. Transient increases in intracellular Ca^2+^ concentration are often early signals of metal stress, triggering the activation of protein kinase-dependent phosphorylation cascades [70].

Mitogen-activated protein kinase (MAPK) signalling pathways are frequently involved in plant responses to heavy metal stress, contributing to signal transduction from membrane-associated sensors to the nucleus. These cascades allow the integration of signals generated by metal stress with those associated with other abiotic stress factors, thus facilitating a coordinated and efficient cellular and molecular response. In mining environments, where plants are simultaneously exposed to multiple stress factors, the interaction between different signalling pathways is essential for effective adaptation.

Although calcium-dependent and MAPK signalling pathways are well characterised under controlled experimental conditions, their long-term dynamics and regulatory stability under conditions of chronic exposure to polymetals remain poorly documented at the ecosystem level [71]. The fluctuating availability of metals and concomitant abiotic stressors in mining environments can substantially alter the intensity and duration of signalling compared to simplified laboratory systems.

### 7.4. Interaction Between Metal Stress and Other Responses to Abiotic Stress

Responses to metal stress do not occur in isolation, but interact with signalling networks activated by other abiotic stress factors, such as drought, salinity or extreme temperatures. This interconnection is reflected in the partial overlap of signalling pathways and gene sets activated under different stress conditions, suggesting the existence of common adaptive mechanisms. The cellular redox state plays a central role in mediating this interaction, influencing both stress perception and the magnitude of molecular responses [72].

In mining environments, where metal stress is rarely singular, the ability of plants to integrate multiple signals and flexibly adjust molecular responses is essential for survival. Understanding the interactions between metal stress and other forms of abiotic stress provides important insights into the adaptive mechanisms of plants in complex and variable environmental conditions characteristic of contaminated mining sites.

In ecosystems affected by mining, exposure to metals frequently occurs alongside water deficit, nutritional imbalance and salinity, leading to overlap and potential competition between signalling networks. Although the mechanisms of interaction between stresses have been analysed in detail under controlled laboratory conditions, their hierarchical integration under chronic field exposure conditions remains insufficiently clarified [73]. Determining how combined stress signals are prioritised and integrated at the transcriptional level in mining environments is an important direction for future research.

To facilitate systematic comparison between controlled experimental evidence and field validation, the strength and contextual support of the main molecular mechanisms discussed in Section 3, Section 4, Section 5 and Section 6 are summarised in Table 6.

Although the molecular signalling pathways underlying responses to heavy metals are extensively characterised in controlled experimental systems, their long-term ecological stability and functional relevance under mining conditions remain incompletely understood [74]. Chronic exposure can drive adaptive transcriptional reprogramming at the population level, potentially involving epigenetic modulation, selection of tolerant genotypes, and stable changes in regulatory network architecture. However, longitudinal field studies integrating transcriptomic profiling with geochemical characterisation of mining sites remain rare. Addressing this gap is essential for translating molecular signalling frameworks into ecological interpretations of plant adaptation in contaminated environments.

## 8. Connecting Mining-Affected Environments and Plant Molecular Responses

Understanding plant molecular responses to heavy metal stress requires integrating mechanistic knowledge gained in the laboratory with the environmental heterogeneity characteristic of mining-affected ecosystems. In these environments, fluctuating geochemical parameters, exposure to polymetals, and concomitant abiotic stressors collectively reshape stress perception, signal hierarchy, and long-term transcriptional stability, thus limiting the direct extrapolation of simplified experimental models to chronically contaminated field conditions.

The comparative framework presented in Table 7 illustrates that controlled experimental exposure mainly captures early signalling events and transient molecular activation, while mining environments favour stable and adaptive reconfiguration of molecular networks. This distinction highlights the importance of differentiating between acute stress signalling and long-term molecular tolerance when interpreting plant responses in chronically contaminated ecosystems. Integrating environmental context into molecular interpretation requires coordinated geochemical profiling of the soil—including metal speciation, pH dynamics, redox fluctuations, and ion competition—alongside transcriptomic and physiological analyses [25]. Only through such simultaneous environmental and molecular characterisations can mechanistic models derived from the laboratory for chronic polymetal exposure scenarios be robustly validated and refined.

Furthermore, differences observed between plant species or populations in distinct mining environments suggest the existence of local adaptations, reflected at the molecular level by variations in gene expression patterns, detoxification efficiency, and redox homeostasis regulation [4]. Analysis of these variations can provide important clues about the factors that determine tolerance to metal stress and facilitate the identification of traits associated with long-term adaptation to contamination.

Field investigations of metallophytic species such as Arabidopsis halleri and Noccaea caerulescens have revealed stable population-specific transcriptional profiles and an increased capacity for metal efflux associated with prolonged exposure to mining substrates [17]. These findings suggest that chronic contamination may drive the selective strengthening of certain regulatory and transport networks, resulting in ecologically stabilised molecular phenotypes rather than transient stress responses.

Persistent metal contamination can act as an environmental filter that selectively stabilises specific molecular traits associated with sustained adaptation to contamination. Under conditions of persistent exposure, regulatory networks associated with efficient detoxification, redox resistance, and controlled metal translocation may be preferentially maintained in plant populations inhabiting mining sites. This filtering process highlights the evolutionary and ecological dimension of molecular adaptation beyond short-term physiological adjustment.

By correlating molecular data with the specific characteristics of real-world environments affected by mining, a more comprehensive and robust picture of plant responses to heavy metal stress emerges, transcending the limitations of isolated laboratory studies [19]. This integrative perspective is essential for developing effective phytoremediation strategies, realistically assessing ecological risks, and understanding the long-term impact of mining activities on terrestrial ecosystems affected by metal contamination.

## 9. Gaps in Knowledge and Future Research Directions

Despite significant progress in understanding the molecular mechanisms underlying plant responses to heavy metal stress, there are still significant gaps in knowledge, particularly in the context of mining-affected environments. One of the major limitations of current research is the discrepancy between molecular studies conducted under controlled experimental conditions and the complexity of field environments, where plant exposure is chronic, multifactorial, and strongly influenced by geochemical variability. This discrepancy hinders the extrapolation of laboratory results to real ecosystems and limits the accurate assessment of the ecological and functional relevance of the molecular mechanisms described [10].

A major gap is the lack of integrated studies that systematically correlate environmental characteristics—such as metal speciation, pH, redox potential, and organic matter content—with the molecular and cellular responses of plants. Although these variables are recognised as key factors controlling metal bioavailability, they are rarely explicitly incorporated into the interpretation of molecular data. The development of experimental approaches that combine detailed characterisation of the environment with molecular analyses represents a critical research direction for reducing this gap.

Another insufficiently explored area concerns the long-term adaptation of plants to chronic metal stress, characteristic of mining sites. Most studies focus on early responses or short-term exposures, while the molecular mechanisms involved in stable tolerance and adaptation at the population level remain poorly understood [66]. Investigating persistent transcriptional changes, long-term metabolic adjustments, and potential epigenetic mechanisms involved in the stabilisation of metal stress tolerance represents a promising and under-explored direction for future research in plant molecular biology.

The integration of multi-omic approaches (transcriptomics, proteomics, and metabolomics) in the study of metal stress offers significant opportunities for gaining a more comprehensive and integrated understanding of the molecular networks involved. Applying these techniques in real environmental contexts, using contaminated soils from mining sites, could allow the identification of molecular signatures specific to complex metal stress that cannot be detected in simplified experimental systems. Furthermore, correlating multi-omic datasets with environmental geochemical parameters would facilitate more robust and ecologically relevant functional interpretations of plant responses [34].

Furthermore, the variability of molecular responses among plant species and populations suggests the presence of local adaptation mechanisms, reflected at both the molecular and physiological levels. Comparative studies of plants from different mining sites could provide valuable information on the factors determining heavy metal tolerance and contribute to the identification of molecular traits useful for species selection in phytoremediation programmes. In this sense, integrating fundamental research with environmental applications is a strategic direction for sustainable development and ecological restoration of areas affected by mining.

From an applied perspective, the mechanistic insights discussed in this review have direct implications for phytoremediation strategies in mining-affected environments. Molecular traits such as restricted translocation from root to shoot, enhanced vacuolar sequestration, efficient antioxidant regulation, and stable transcriptional reprogramming under conditions of chronic metal exposure are particularly relevant to phytostabilisation approaches [67]. In contrast, mechanisms associated with controlled long-distance transport and metal chelation can be exploited in phytoextraction strategies, albeit with increased ecological risk. It is important to note that the effectiveness of phytoremediation cannot be reliably predicted based solely on molecular responses identified under laboratory conditions, highlighting the need to integrate molecular screening with site-specific environmental assessment.

To address these persistent knowledge gaps in a structured manner, future research efforts should be organised on complementary temporal and methodological scales.

 Short-term priorities include:

• Systematic field validation of key metal transporters (ZIP, HMA, NRAMP) in plants naturally growing on mining sites;
• Direct in situ assessment of intracellular ROS dynamics under fluctuating geochemical conditions;
• Integration of soil geochemical profiling (metal speciation, pH, redox potential, competitive ion interactions) with molecular and transcriptomic analyses.

Medium-term research directions should focus on:

• Longitudinal transcriptomic monitoring of chronically exposed populations;
• Applying integrated multi-omic approaches in field-relevant exposure scenarios;
• Investigation of epigenetic mechanisms contributing to the stabilisation of metal tolerance;
• Quantifying the energy trade-offs associated with sustained detoxification and antioxidant activity.

Long-term strategic objectives include:

• Development of predictive molecular markers for assessing the suitability of phytostabilisation and phytoextraction;
• Incorporation of molecular stress indicators into ecological risk assessment frameworks;
• Establishing interdisciplinary monitoring systems that link molecular diagnosis of plants with environmental geochemistry.

The shift from mechanistic description to ecology-based interpretation requires a transition from isolated laboratory experiments to integrative, field-based molecular ecology. Strengthening the interface between plant molecular biology and environmental sciences will be essential for translating mechanistic knowledge into sustainable strategies for the management and restoration of ecosystems affected by mining.

## 10. Conclusions

Heavy metal contamination in environments affected by mining represents a complex abiotic stress context, characterised by chronic, polymetallic exposure and high geochemical variability. Under these conditions, plant responses cannot be interpreted exclusively through the mechanisms described in simplified experimental systems, but must be analysed in an integrative framework that correlates molecular processes with site-specific environmental parameters.

The current literature demonstrates that metal tolerance results from the coordinated integration of selective absorption and transport, redox homeostasis regulation, detoxification mechanisms (chelation, compartmentalisation, apoplastic immobilisation) and gene expression reprogramming. However, for most mechanisms extensively characterised in the laboratory, robust validation under conditions of chronic polymetallic exposure remains insufficiently systematised.

A central aspect highlighted in this analysis is the role of geochemical variability—pH, redox potential, metal speciation and multimetallic interactions—as direct modulators of molecular regulation. Thus, metal stress in mining environments must be understood as a dynamic process in which oxidative pressure, the energy costs of detoxification, and metabolic trade-offs influence the long-term stability of tolerance.

From an applied perspective, the differentiation between translocation restriction and hyperaccumulation strategies has direct implications for the design of phytostabilisation or phytoextraction interventions. However, the effectiveness of these approaches cannot be deduced solely from mechanistic laboratory data and requires the integration of molecular assessment with geochemical and ecological analysis of contaminated sites.

Future research should prioritise in situ molecular investigations, comparative approaches between adapted and non-adapted populations, and longitudinal studies on redox dynamics and transcriptional regulation under real mining conditions. Such interdisciplinary integration is essential to transform mechanistic knowledge into predictive tools and sustainable solutions for the restoration of ecosystems affected by mining activities.

## Figures and Tables

**Table 1:** Major heavy metals associated with mining activities and their primary molecular and cellular effects in plants.

Heavy Metal	Typical Mining Sources	Dominant Mobility Processes	Major Cellular And Molecular Targets	Predominant Physiological and Biochemical Effects
Cd	Mine tailings, tailings ponds, flotation residues	High mobility under acidic conditions; weak retention by solid phases	Plasma membrane, cytosol, vacuole; binding to phytochelatins	Severe oxidative stress, inhibition of photosynthesis, disruption of essential nutrient uptake
Pb	Sulphide-rich mining wastes, contaminated dust	Low mobility; predominant accumulation in roots	Cell wall, plasma membrane, apoplast	Inhibition of root elongation, altered membrane permeability, interference with Ca metabolism
Zn	Mine tailings, acid mine drainage	Moderate mobility; influenced by pH and organic matter	Cytosol, metalloenzymes, nucleus	Redox imbalance, enzyme activity disruption, metabolic stress at excessive concentrations
Cu	Processing residues, tailings ponds	Redox-dependent mobility; active in redox cycling	Chloroplasts, mitochondria, redox enzymes	ROS generation, impairment of photosystems, inhibition of cellular respiration
Ni	Mine tailings, exposed ultramafic rocks	Variable mobility; increased under acidic conditions	Cytosol, nucleus, nitrogen metabolism-related enzymes	Disruption of nitrogen metabolism, growth inhibition, oxidative stress
Cr (VI)	Industrial–mining wastes, oxidation of Cr(III)	High mobility under oxidising conditions	Cytosol, nucleus, DNA	Genotoxicity, inhibition of gene expression, severe oxidative stress
As	Mine tailings, acid mine drainage	Redox- and pH-dependent mobility	Cytosol, vacuole, phosphate transport systems	Interference with phosphorus metabolism, growth inhibition, oxidative stress

**Table 2:** Comparative characteristics of the main families of heavy metal transporters in acute and chronic exposure contexts.

Family of Transporters	Preferred Substrates	Subcellular Localisation	Expression Pattern Under Acute Experimental Stress Conditions	Evidence in Conditions of Exposure Chronic in Mining Environments
ZIP (ZRT/IRT-type proteins)	Zn^2+^, Fe^2+^, Mn^2+^, Cd^2+^	Plasma membrane	Rapid expression Inducible in response to metal excess	Limited direct validation in the field; differential expression inferred in metallophyte populations
NRAMP (natural resistance-associated macrophage proteins natural resistance)	Fe^2+^, Mn^2+^, Cd^2+^	Plasma plasma; endomembrane system	Inducible upon exposure to a single metal	Partial evidence from field populations exposed to polymetals
HMA (heavy metal ATPases)	Zn^2+^, Cd^2+^, Cu^+^	Plasma membrane; tonoplast	Inducible and dependent of activity	Stable overexpression of HMA4 documented in *Arabidopsis halleri* populations from Zn-rich mining soils
ABC transporters (selected members)	Metal chelate complexes	Tonoplast	Inducible under acute stress conditions	Validation limited quantitative field validation; involved in chronic vacuolar sequestration chronic

**Table 3:** Strength of evidence and status of environmental validation of the main molecular mechanisms involved in the adsorption and transport of heavy metals.

Molecular Mechanism	Laboratory Laboratory	Pot Experiments	Field Validation in Affected by Mining Operations	The Context of Exposure Validated	Examples Representative Field Examples
ZIP conveyors	Extensive	Limited	Validation direct limited; inferred in the case of metallophytes	Dominant acute	Expression ZIP Differentiated in *A. halleri* populations
NRAMP transporters	Extensive	Moderate	Limited	Acute/partial chronic	Population-specific expression patterns reported in metallophytes
HMA transporters	Extensive	Moderate	Documented in hyperaccumulators	Chronic exposure to Zn/Cd	HMA4 overexpression in *A. halleri* (field populations)
Translocation of metals in xylem/phloem	Strong	Moderate	Documented in hyperaccumulator species	Chronic	Improved transport from root to shoots in *N. caerulescens*
Vacuolar sequestration (ABC transporters)	Strong	Moderate	Partial evidence	Chronic adaptation	Field populations exhibiting stable intracellular metal storage
Chelation mediated by phytochelatin	Strong	Moderate	Documented in metallophytes	Acute and chronic	High PC levels in plants exposed in the field
Metallothioneins	Strong	Moderate	Reported in tolerant populations in the field	Acute; possibly chronic	Upregulation of MT gene in metal-adapted ecotypes
Restriction of translocation from root to shoots	Moderate	Moderate	Well-documented in metallophytes phytostabilisers	Chronic	Root retention strategies in vegetation in areas settling areas

**Table 4:** Strength of evidence for redox and antioxidant mechanisms in different exposure contexts.

Mechanism	Validation in Laboratory Studies	Validation in Soil/Pot Experiments	Validation in Field (Mining Sites)	Type of Exposure Validated
ROS overproduction	Extensive (direct measurement of ROS accumulation)	Moderate	Limited (often indirect biomarkers)	Predominantly acute exposure
SOD/CAT activation	Extensive	Moderate	Variable evidence	Acute and partially chronic
Glutathione dynamics	Extensive	Moderate	Reported in metallophytes	Acute and chronic exposure
Redox signalling cascades (MAPK, Ca^2+^)	Extensive	Limited	Rare direct validation	Mainly acute exposure
Long-term shifts in redox threshold	Emerging evidence	Limited	Hypothesised; limited field validation	Chronic exposure

**Table 5:** Key molecular detoxification mechanisms contributing to heavy metal tolerance in plants.

Molecular Mechanism	Key Components	Location Primary Cellular	Functional Role in Metal Tolerance	Integration with Other Processes
Intracellular chelation	Phytoquelatins, metallothioneins	Cytosol, vacuole	Binding of free metal ions and reduction in cytosolic toxicity	Facilitates vacuolar sequestration; energy-dependent synthesis; strongly influenced by redox state
Metal transport and compartmentalisation	HMA, ABC, CAX transporters	Plasma membrane, tonoplast	Active sequestration of metals in vacuoles or exclusion from sensitive tissues	ATP-dependent transport; coordinated with gene expression gene and chelator availability
Regulation of redox homeostasis	SOD, CAT, APX, glutathione	Cytosol, chloroplasts, mitochondria	Limitation of metal-induced ROS accumulation and maintenance of redox balance	Closely linked to stress signalling; requires sustained metabolic support
Modulation of gene expression	Factors of Transcription factors, stress-responsive genes	Nucleus	Induction coordinated induction of detoxification and tolerance pathways	It integrates redox signals, the state of intracellular metals and environmental signals
Apoplastic immobilisation	Cell wall components, pectins	Apoplast	Partial immobilisation of metals at the extracellular level	Energy-conserving mechanism; reduces cellular influx of metals
Metabolic reprogramming	Adjustments of energy metabolism and nitrogen	Cytosol, mitochondria	Maintaining metabolic functionality under of prolonged metal stress	Supports antioxidant and detoxifying efficiency; reflects energy compromises
Phenotypic plasticity	Adaptive responses at the tissue and organ level	Whole plant	Adjustment of growth patterns growth and architecture Architecture of the plant	The emerging result of molecular detoxification

**Table 6:** Strength of evidence and validation status of key molecular mechanisms in different exposure contexts.

Mechanism	Validation in the Laboratory	Pot Experiments	Field Validation (Mining Sites)	Context of Exposure Predominantly Validated
ZIP transporters	Extended functional characterisation in model species (e.g., *Arabidopsis thaliana*)	Limited validation in controlled soil systems	Confirmation sporadic direct confirmation in the field; inferred from expression analyses in metallophyte populations chronically exposed to metals	Acute systems, predominantly with a single metal
NRAMP transporters	Extensive molecular and functional studies	Moderate validation semi-controlled exposure conditions semi-controlled exposure conditions soil	Limited direct quantification in the field; indirect evidence from metallophytes exposed to polymetallic mining substrates Acute exposure; partial evidence	Acute exposure; partial evidence in chronic conditions
HMA carriers	Extensive mechanical characterisation Extensive mechanical, including overexpression studies	Moderate validation in soil-based experiments	Stable overexpression of HMA4 documented in *Arabidopsis halleri* populations living in Zn-rich mining soils; improved translocation from root to shoot observed under natural exposure conditions	Controlled systems; validated under chronic conditions of Zn/Cd-rich soil
Synthesis of phytochelatin	Biochemical validation and extensive genetic validation	Moderate confirmation in experiments on contaminated soils	Increased phytochelatin accumulation reported in metallophytes collected in the field under conditions of chronic exposure to polymetals	Acute and chronic exposure contexts
Vacuolar sequestration	Extensive studies on the tonoplast transporter and chelation	Moderate validation in systems of soil exposure	Evidence of sustained intracellular compartmentalisation of metals in metallophytes colonising mining residues; limited long-term quantitative field studies	Contexts of chronic adaptation
ROS signalling	Extensive mechanical dissection in acute exposure models	Limited validation under semi-controlled conditions	Limited direct in situ quantification; mainly indirect field evidence through profiling antioxidant enzymes in populations in mining areas	Laboratory predominantly acute laboratory systems
Transcriptional reprogramming	Extensive transcriptomic characterisation under exposure conditions short-term	Moderate validation in based on soil	Emerging evidence of stable population-specific transcriptional profiles in metallophytes from mining areas	Chronic exposure and adaptive contexts

**Table 7:** Key conceptual differences between controlled experimental metal exposure and chronic exposure in mining-impacted environments.

Characteristic	Controlled Experimental Exposure	Chronic Exposure in Mining-Impacted Environments
Exposure duration	Short-term (hours–days), well defined	Long-term (months–years), continuous
Metal composition	Typically a single metal or simple combinations	Complex mixtures of heavy metals and metalloids
Concentrations	Constant, strictly controlled	Spatially and temporally variable
Chemical speciation	Stable and well characterised	Dynamic, dependent on pH, redox potential, and weathering processes
Geochemical conditions	Homogeneous and reproducible	Heterogeneous and fluctuating (pH, redox, moisture)
Type of stress	Predominantly acute	Predominantly chronic
Dominant molecular responses	Early signalling and rapid activation of stress-responsive genes	Stable and adaptive transcriptional reprogramming
Metabolic regulation	Transient adjustments	Long-term metabolic adjustments
Cellular compartmentation	Limited and often incompletely expressed	Efficient, long-term sequestration strategies
Phenotypic plasticity	Low or absent	Pronounced, with structural and functional adaptations
Ecological relevance	High for fundamental mechanisms	High for adaptation, tolerance, and survival
Predictive capacity for field conditions	Limited	Directly relevant to real-world conditions

## Data Availability

No new data were created or analyzed in this study.

## Abbreviations

The following abbreviations are used in this manuscript:

ABC	ATP-binding cassette (transporter)
APX	Ascorbate peroxidase
ATP	Adenosine triphosphate
Ca^2+^	Calcium ion
CAT	Catalase
CAX	Cation/H^+^ exchanger
DNA	Deoxyribonucleic acid
Eh	Redox potential
H_2_O_2_	Hydrogen peroxide
HMA	Heavy metal ATPase
HMAs	Heavy metal ATPases
MAPK	Mitogen-activated protein kinase
MT	Metallothionein
MTs	Metallothioneins
NRAMP	Natural Resistance-Associated Macrophage Protein
OM	Organic matter
PC	Phytochelatin
PCs	Phytochelatins
POD	Peroxidase
ROS	Reactive oxygen species
SOD	Superoxide dismutase
TF	Transcription factor
ZIP	ZRT/IRT-like protein
